# Supplementary material for: Transcatheter Management of Pulmonary Sequestrations in Children—A Single-Center Experience
Source: Children (Basel). 2023 Jul 10;10(7):1197. doi: 10.3390/children10071197 (PMC10378210; doi:10.3390/children10071197)
Supplement: Supplementary file 1 [file children-10-01197-s001.zip › children-2405287-supplementary.pdf]

Table S1. Associated cardiac defects.

	N (%)
**Associated cardiac defects*, n=61**	
**Associated anomalous pulmonary vein into the systemic venous system**	47 (77)
Atrial septal defect	30 (49)
Ventricular septal defect	20 (32)
Persistent arterial duct	15 (32.8)
Left superior caval vein	10 (24.6)
Aortic coarctation	4 (6.6)
Bicuspid aortic valve	2 (3.3)
Pulmonary vein stenosis	2 (3.3)
Atrioventricular canal defect	2 (3.3)
Others	6 (9.83)
